# Pilot safety evaluation of doxorubicin chemotherapy combined with non-specific immunotherapy (Immunocidin®) for canine splenic hemangiosarcoma

**DOI:** 10.1371/journal.pone.0279594

**Published:** 2022-12-22

**Authors:** Margaret L. Musser, Giovanna M. Coto, Yuan Lingnan, Jonathan P. Mochel, Chad M. Johannes

**Affiliations:** 1 Veterinary Clinical Sciences, Iowa State University, Ames, Iowa, United States of America; 2 Biomedical Sciences, Iowa State University, Ames, Iowa, United States of America; 3 SMART Pharmacology, Iowa State University, Ames, Iowa, United States of America; University of Bologna, ITALY

## Abstract

Canine splenic hemangiosarcoma (HSA) is an aggressive tumor with a short overall survival time (OST) despite treatment with splenectomy and adjuvant doxorubicin. Modulation of the immune system has been shown to be effective for a variety of human tumors, and may be effective for canine tumors, including HSA. Immunocidin® is a non-specific immunotherapy based on a mycobacterial cell wall fraction. Preliminary work suggests Immunocidin® is safe to give intravenously (IV) in tumor-bearing dogs. This work aimed to evaluate the safety of doxorubicin and Immunocidin® combination in dogs with naturally occurring splenic HSA. A secondary aim of this study was to collect preliminary efficacy data to support a subsequent comprehensive, prospective clinical trial in canine patients with HSA, if the combination of doxorubicin and Immunocidin® was found to be safe. Eighteen dogs with stage II-III splenic HSA were recruited to receive 5 doses of sequential IV doxorubicin and Immunocidin® at two-week intervals following splenectomy. Adverse events (AEs) were graded according to the Veterinary Cooperative Oncology Group v1.1 (VCOG) scheme. Overall survival time was calculated from the date of splenectomy to date of death or loss to follow-up. AEs during administration were infrequent, the most common being hypertension. One patient developed limb and facial twitching and was removed from the study. After infusion, common AEs included lethargy, hyporexia, and diarrhea. One patient developed VCOG grade 5 diarrhea, thrombocytopenia, and anemia. Modifications in the treatment regimen were made to prevent these signs in subsequent patients. The median OST in dogs treated with the combination therapy was estimated at 147 days (range: 39–668 days). Although generally safe, the combination of doxorubicin and Immunocidin® appeared to cause more gastrointestinal effects than doxorubicin alone, and no apparent improvement in OST was noted in this population of dogs.

## Introduction

Canine splenic hemangiosarcoma (HSA) is a devastating malignancy that typically affects older, large breed dogs [1]. Overall survival times (OST) following surgery alone have been reported to range from 1–3 months [1, 2], while treatment with splenectomy and adjuvant chemotherapy range from 4–8 months [2–8], depending on the clinical stage of disease (typically reported to be shorter in dogs with stage II or III disease compared to stage I, although the literature is mixed) [7–10]. Given this dismal outcome, multiple alternative treatment approaches have been investigated, including various forms of immunotherapy.

An early study evaluating various treatments for dogs with HSA suggested that the longest survival times were achievable when treatment with surgery, bacterial immunotherapy, and chemotherapy were pursued [11]. Given the initial success of immunotherapy, subsequent studies evaluated the immunomodulator liposome-encapsulated muramyl tripeptide phosphatidylethanolamine (L-MTP-PE) in combination with intravenous (IV) doxorubicin and IV cyclophosphamide [12]. Specifically, dogs that received IV L-MTP-PE and chemotherapy had a significantly longer disease-free interval (DFI) and OST compared to those that received placebo and chemotherapy [12]. This study also suggested that L-MTP-PE decreased the development of metastasis, as those with stage II disease developed metastasis in fewer organs and less frequently in the liver [12].

Given the early success with L-MTP-PE, which is no longer available in the United States, two therapeutic vaccines have been evaluated in dogs with HSA: a novel cationic liposome and DNA complex (LDC) vaccine [13], and a dendritic cell vaccine [14]. LDCs have been shown to be potent activators of innate immunity and antitumor activity in mice [15, 16], and were successful at inhibiting tumor angiogenesis and resulted in tumor regression in some dogs with soft tissue sarcomas [17]. In dogs with HSA of various anatomical locations, including the spleen, LDC in combination with doxorubicin elicited strong humoral immune responses against a control antigen and antibody responses against canine HSA cells. However, in those dogs with splenic HSA, OST was not significantly impacted [13]. Similarly, investigations with a dendritic cell vaccine plus low-dosage doxorubicin (20 mg/m^2^) following splenectomy in dogs with confirmed HSA was shown to be safe but did not improve OST [14].

Because of the bleak response to chemotherapy alone in canine HSA, the addition of immunotherapy to standard treatment continues to be an appealing approach. Immunocidin® is a mycobacterial cell wall fraction, non-specific immunotherapeutic, USDA-licensed for the intratumoral treatment of mixed mammary tumors and mammary adenocarcinomas in dogs [18]. Immunocidin® has immunomodulatory properties through activation of immune system receptors, increased cytokine production by monocytes, macrophages, and dendritic cells, increased colony stimulating factor production, and the induction of innate immune responses and cell-mediated immunity [18]. Preliminary evidence suggests it is safe to give IV in a variety of species [19–23], and in combination with multiple chemotherapeutics in dogs and cats, including doxorubicin [18]. Initial evidence in three dogs also suggested there may be efficacy specifically in canine HSA [18]. Therefore, the aims of this study were to further evaluate the safety of combining maximum tolerated dose doxorubicin and Immunocidin® when given IV to dogs with splenic HSA. Upon demonstration of safety of this novel combination, a secondary aim was to collect preliminary efficacy data, compared to a historical control group of dogs treated with doxorubicin alone, to support a subsequent comprehensive, prospective clinical trial in canine patients with HSA.

## Materials and methods

### Recruitment strategy and inclusion criteria

Patients were recruited to this pilot study if they had met the following criteria: dogs had splenectomy with histologically confirmed stage II-III splenic HSA, with a modified Eastern Comparative Oncology Group (ECOG) constitutional performance score of 0–1 at enrollment [24]. Exclusion criteria included the following: evidence of a serious concomitant disorder (e.g., clinically significant cardiac disease that would preclude the use of doxorubicin), diagnosis of a concurrent malignancy, previous chemotherapy, steroids, or immunotherapy (including lokivetmab and oclacitinib) for any reason within 4 weeks preceding enrollment. Patients were recruited to study sites with a board-certified oncologist. The protocols for this study were approved by the Animal Care and Use Committee at Iowa State University (IACUC-18-224). In addition, written, informed consent was provided to each owner and a signature was required prior to enrollment in the study. Following splenectomy, all masses were histologically confirmed to be HSA by multiple pathologists, and a mitotic index was reported. At enrollment, prior to the first doses of doxorubicin and Immunocidin®, patients were required to have a CBC, chemistry panel, and chest radiographs. Blood work was also required the day of treatment; chest radiographs were accepted if completed within 2–3 weeks of enrollment (Eg. at initial ER visit). Abdominal imaging was not required at initial presentation as the diagnostic value of abdominal ultrasound and/or computed tomography to confirm malignant disease in the spleen [25, 26] and liver is limited [26]. Similarly, abdominal imaging was not required at study enrollment as patients had just undergone complete exploratory surgery.

### Treatment protocol

Following splenectomy, dogs enrolled in the study were started on standard treatment (IV doxorubicin) followed immediately with IV Immunocidin®. Prior to infusion, dogs were pretreated with diphenhydramine (2 mg/kg IM) and ondansetron (0.5 mg/kg IV). Initially, dogs received doxorubicin (30 mg/m^2^ IV for those >15 kg; 1 mg/kg for those ≤15 kg) followed by Immunocidin®. Immunocidin® was supplied as an oil emulsion containing 1 mg/mL of the mycobacterial cell wall fraction formulated with phosphate buffered saline and squalene. Dosage was based on weight, as previously reported: 0.1 mg for patients <5 kg, 0.25 mg for patients 5–10 kg, 0.3 mg for patients between 10–15 kg, 0.5 mg for patients 15–25 kg, 0.75 mg for patients 25–35 kg, and 1 mg for patients >35 kg [21]. Each dose was diluted in 75 mL of sterile 0.9% NaCl and administered IV over 30 minutes (personal recommendation from Novavive). Prior to the Immunocidin® infusion, patients’ temperature, pulse, respiratory rate, and blood pressure were recorded. These parameters were evaluated again at the end of the infusion period, and every 30 minutes following the infusion for 2 hours. Following treatment, patients were prescribed maropitant (2 mg/kg PO q 24 hours) for 4–5 days. This regimen was repeated every 2 weeks for a total of 5 treatments.

The fourth patient enrolled in the study experienced severe adverse events (AEs), including Veterinary Cooperative Oncology Group (VCOG) grade 5 diarrhea, thrombocytopenia, and anemia. The patient ultimately died, although the exact cause of death was unknown. An informal mid-study analysis of AEs was conducted, and it appeared that clinically patients were exhibiting more gastrointestinal (GI) effects with the combination of Immunocidin® and doxorubicin than would be expected with doxorubicin alone. Out of an abundance of caution, the treatment regimen was amended: doxorubicin dosage was decreased by 10% (27 mg/m^2^ for those >15 kg; 0.9 mg/kg for those ≤15 kg), maropitant (1 mg/kg IV) was added to the pretreatment regimen, and ondansetron (0.5–1 mg/kg PO q 12 hours x 4 days), metronidazole (10 mg/kg PO q 12 hours) or tylosin (7–15 mg/kg PO q 12–24 hours) to be started on the first day of clinically soft stools, and capromorelin (3 mg/kg PO q 24 hours x 4 days) as needed to simulate appetite, were added to the supportive regimen following infusion. Further chemotherapy treatment delays and dosage adjustments were allowed at the discretion of the attending oncologist. Immunocidin® was not decreased in dosage.

Whole blood samples for a complete blood count were collected the day of each treatment, and 7–10 days following at least the first two infusions, to ensure a safe dosage combination was attained. Clinical chemistry analysis was performed at the discretion of the attending oncologist. Additional supportive care measures (subcutaneous or IV fluids, antibiotics, antidiarrheals, and appetite stimulants) were allowed at the discretion of the attending oncologist. No Yunnan Baiyao, I’m Yunity®, therapeutic vaccinations, steroids, non-steroidals, adrenocorticotropic hormone, or other immunotherapies (including lokivetmab and oclacitinib) were allowed while on trial. Restaging was at the recommendation and discretion of the attending oncologist.

Presenting blood work was evaluated for previously identified prognostic factors (thrombocytopenia and anemia) [27–29]. Thrombocytopenia was defined as an absolute platelet count <100,000/uL, and anemia as a hematocrit <32%, as previously described [30]. Adverse events were graded according to the VCOG v1.1 grading scale [31]. All case information was collected via an electronic data capture platform (REDCap, Vanderbilt University, Nashville, TN, USA). The median OST was calculated from the date of splenectomy to date of death or loss to follow-up.

### Statistical analysis

All statistical analyses were performed in R (v4.0.3) using the *survminer* package (v0.4.9). Median OST was derived with a 95% CI. Patients still alive at data capture were censored at the last date of contact. Median OST was compared to historical controls treated with surgery and single agent doxorubicin [8, 32]. The effect of the following variables: (i) anemia (hematocrit <32%), (ii) thrombocytopenia (platelet count <100,000/L) and (iii) mitotic index (<11 mitoses/10 hpf vs ≥ 11 mitoses/ 10 hpf) [5, 29] on the median OST was evaluated using a combined log rank test and Cox proportional hazard model, verifying assumptions of proportional hazard ratio by using Schoenfeld residuals and hypothesis testing between covariates and log(time). Evaluation of the impact of clinical stage on OST was not conducted due to the small number of stage III patients. Model variables were further dichotomized into “low” vs. “high” categories using their median value. Kaplan Meier curves were generated in R (v4.03) for visualization. P-values < 0.05 were considered as statistically significant. A power analysis for a type 1 error α of 0.05 with power (1-β) = 0.80 was performed in case of inadequate sample size, when appropriate.

## Results

### Demographic and presenting clinical information

Eighteen dogs were prospectively enrolled at 5 oncology practices from March 2019 to November 2020. The median age of enrolled dogs was 9.5 years (range 4.0–13.0), with a median weight of 27.7 kg (range 8.0–42.5 kg). Breeds included Labrador Retriever (n = 5), Boxer (n = 2), Golden Retriever (n = 2), and one each of the following breeds: Collie Mix, Goldendoodle, Great Pyrenees Mix, Hound Mix, Irish Setter, Miniature Schnauzer, Poodle Mix, Shepherd Mix, and Wheaten Terrier. All dogs had a modified ECOG performance score of 0. Two dogs had concomitant diseases including epilepsy controlled with zonisamide and a grade IV/VI heart murmur due to stage B2 myxomatous mitral valve disease treated with pimobendan and benazepril. No dogs received chemotherapy or immunotherapy prior to treatment with doxorubicin and Immunocidin®.

Presenting complaints included lethargy (n = 13), hyporexia (n = 8), abdominal pain (n = 3), weakness (n = 6), distended abdomen (n = 1), vomiting (n = 1), polyuria/polydipsia (n = 1), panting (n = 1), and a suspect seizure at home (n = 1). Two patients were presented for routine wellness examinations and blood work with no clinical signs. An abdominal ultrasound was recommended for one of these patients due to IRIS chronic kidney disease stage 2 identified on routine blood work, and the other due to progressive anemia. Ultrasound examination revealed a 6.6 x 6.8 cm, cavitated, incidental splenic mass in one patient, and a bleeding splenic mass in the second; thus, surgical removal of the spleen was recommended for both patients.

At presentation, all patients had a CBC, chemistry panel, and thoracic radiographs completed for initial staging. Eleven patients had an AFAST (abdominal focused assessment with sonography for trauma, triage, and tracking; n = 4) or full abdominal ultrasound (n = 7) prior to pursuing surgery. Three of the four patients evaluated via AFAST were diagnosed with a hemoabdomen and suspect splenic mass. The fourth patient was diagnosed with a hemoabdomen only. Six of the patients evaluated with a full abdominal ultrasound were found to have a hemoabdomen and splenic mass, with no evidence of metastatic disease. The seventh patient was diagnosed with a large splenic mass but no hemoabdomen. One additional patient had abdominal radiographs prior to surgery that reveled a large abdominal mass. Two other patients had full abdominal ultrasounds after splenectomy with no evidence of metastatic disease. One additional dog had multiple liver nodules noted during surgery. These were biopsied and histopathology was consistent with metastatic hemangiosarcoma. Thus, sixteen dogs had stage II disease based on the size of the splenic tumor or presence of a hemoabdomen, and two dogs were considered to be stage III with confirmed metastasis to the liver (treated in the gross disease setting) [2].

Sixteen dogs (94%) were thrombocytopenic at presentation (one patient did not have a viable platelet count available for analysis; it was reported as adequate), while 9/18 (50%) of dogs were anemic. All dogs were histologically diagnosed with splenic HSA and 13/18 patients had a mitotic index reported per 10 high powered fields (hpf). Seven patients had a mitotic index < 11 mitoses/10 hpf; 6 had a mitotic index ≥ 11 mitoses/10 hpf. All dogs started the treatment regimen at a median of 17 days (range: 8–41 days).

Four dogs were started on the doxorubicin and Immunocidin® protocol within 14 days of splenectomy (range: 8–13 days). The remaining 14 dogs began the treatment regimen a median of 20.5 days following splenectomy (range: 16–41 days). Delays were due to a variety of reasons, most commonly including delayed histopathology, scheduling difficulties, and owner compliance.

### Adverse events

**Fig 1:**
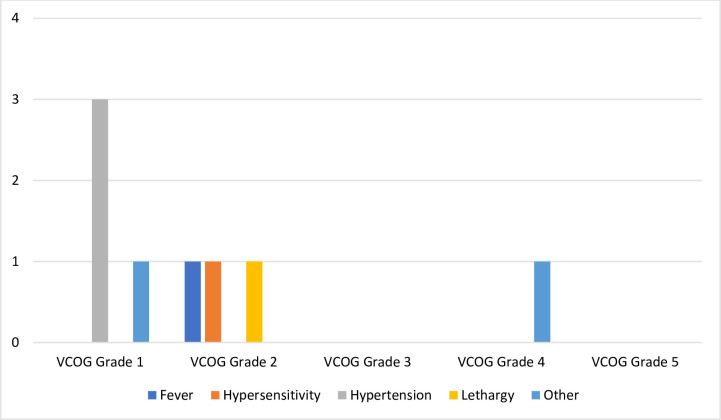
Adverse event grades during and immediately after* Immunocidin® infusion in dogs with splenic hemangiosarcoma. VCOG: Veterinary Cooperative Oncology Group; *Patients were monitored in the hospital for 2 hours after Immunocidin® infusion.

**Fig 2:**
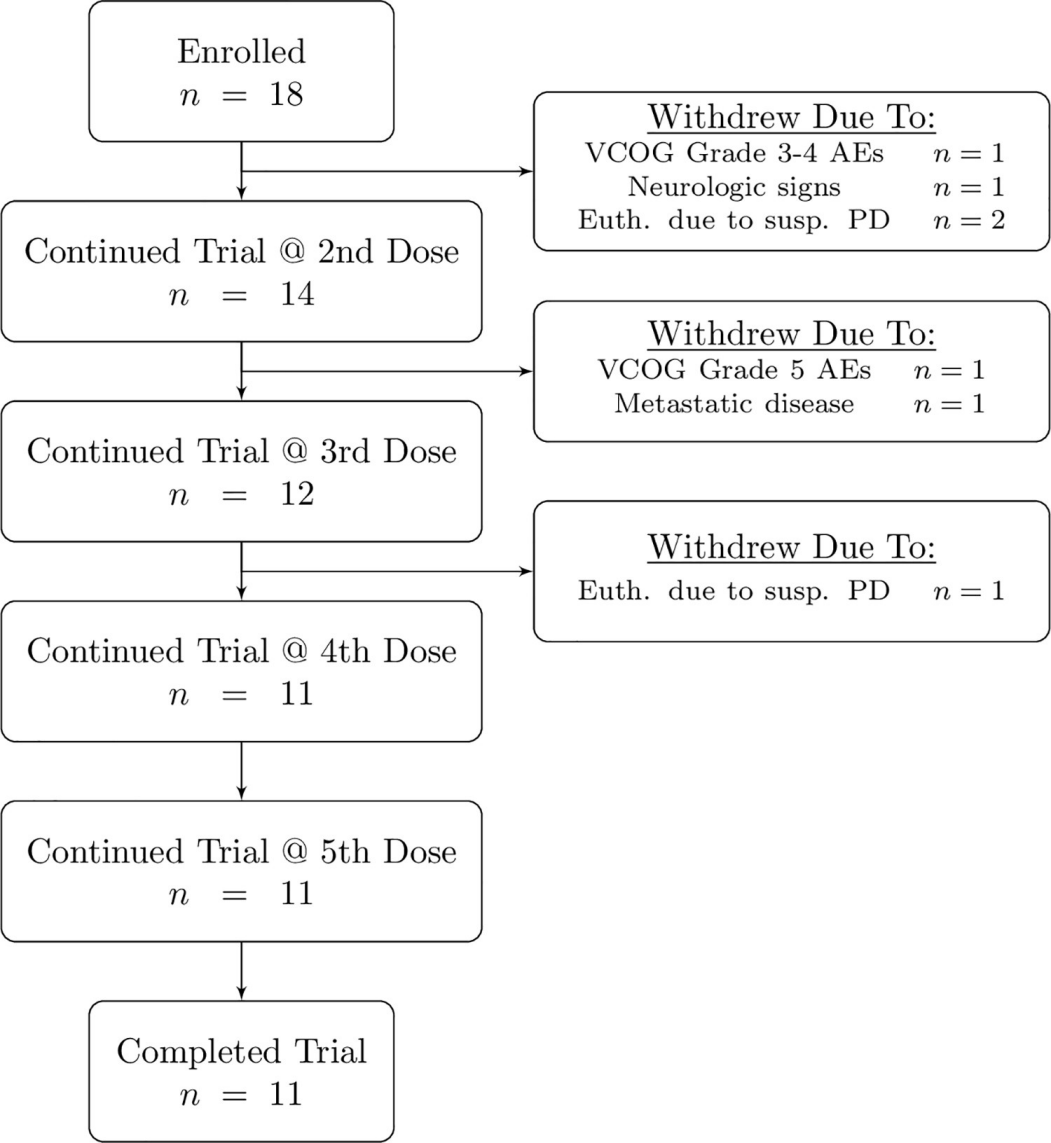
Patient retention for dogs with splenic hemangiosarcoma treated with doxorubicin and Immunocidin®.

Adverse events during administration and up to 2 hours following administration of doxorubicin and Immunocidin® were infrequent and included hypertension, fever, hypersensitivity, and lethargy (Table 1 and Fig 1). One patient developed VCOG grade 1 limb and facial twitching at the end of the first doxorubicin infusion and during the first Immunocidin® infusion. These changes intensified and the Immunocidin® was stopped. The patient returned the following day to finish the Immunocidin® treatment and VCOG grade 1 limb and facial twitching was observed again. Treatment was discontinued and the patient was removed from the study (Fig 2).

**Table 1:** Adverse events noted during and immediately after* infusion of Immunocidin® in dogs with splenic hemangiosarcoma.

AE	During Tx 1 (n = 18)	During Tx 2 (n = 14)	During Tx 3 (n = 12)	During Tx 4 (n = 11)	During Tx 5 (n = 11)	Total
**Hypertension**	0	0	1	1	1	3
**Other**	1	1	0	0	0	2
**Fever**	1	0	0	0	0	1
**Hypersensitivity**	0	1	0	0	0	1
**Lethargy**	0	1	0	0	0	1
**Diarrhea**	0	0	0	0	0	0
**Hypotension**	0	0	0	0	0	0
**Hyporexia**	0	0	0	0	0	0
**Nausea**	0	0	0	0	0	0
**Vomiting**	0	0	0	0	0	0

AE: Adverse event; Tx: treatment

*Patients were monitored in the hospital for 2 hours after Immunocidin® infusion.

**Fig 3:**
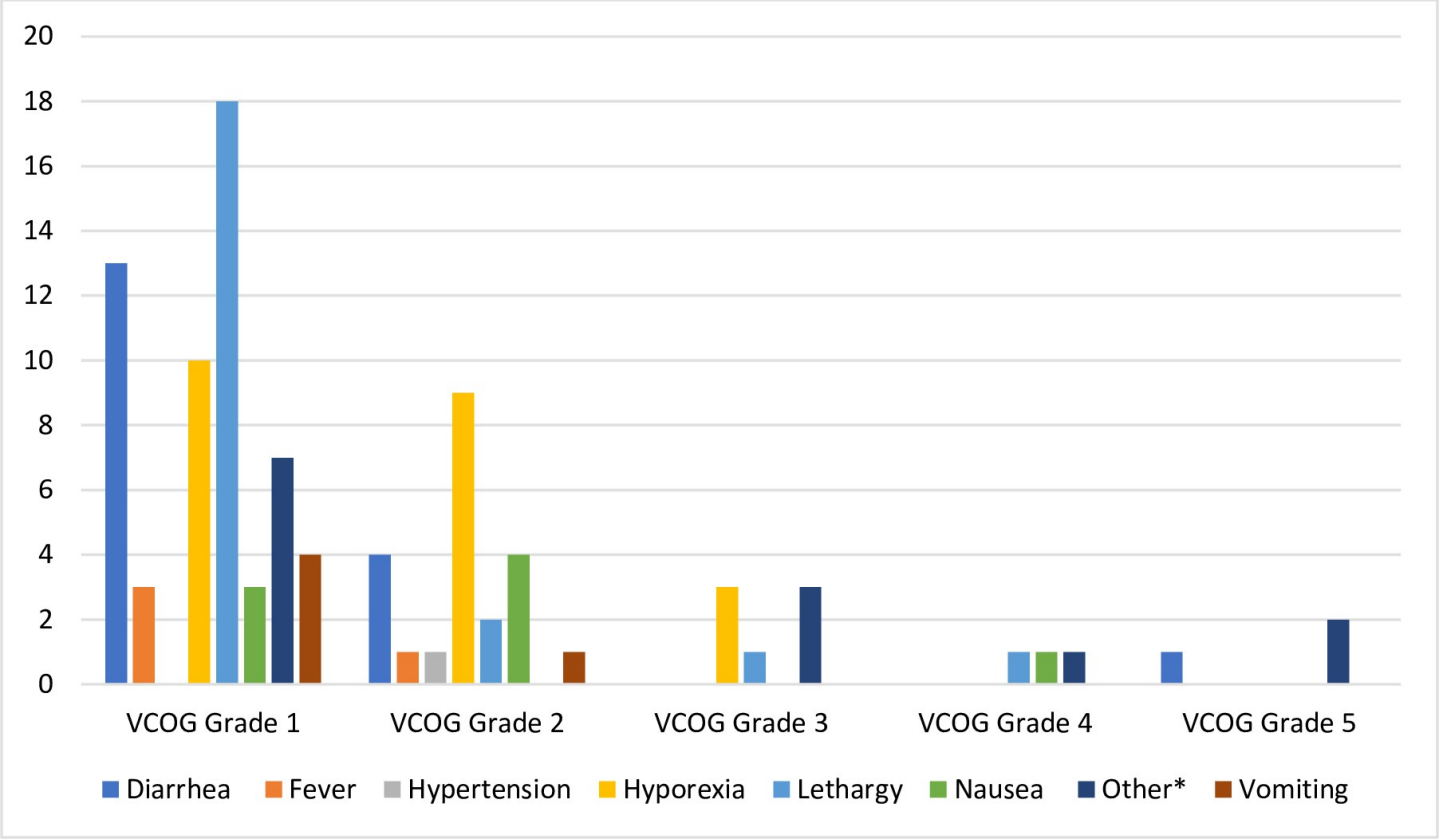
Adverse event grades noted up to two weeks following Immunocidin® infusion in dogs with splenic hemangiosarcoma. VCOG: Veterinary Cooperative Oncology Group; *One episode of proteinuria was not quantified via urine protein/creatinine ratio, and thus not graded.

Most AEs were observed in the two weeks following the sequential infusions of doxorubicin and Immunocidin®. The most common AEs were lethargy, hyporexia and diarrhea (Table 2 and Fig 3).

**Table 2:** Adverse events noted up to two weeks following infusion of Immunocidin® in dogs with splenic hemangiosarcoma.

AE	After Tx 1 (n = 18)	After Tx 2 (n = 14)	After Tx 3 (n = 12)	After Tx 4 (n = 11)	After Tx 5 (n = 11)	Total
**Lethargy**	8	3	5	4	2	22
**Hyporexia**	8	2	6	4	2	22
**Diarrhea**	8	3	2	3	2	18
**Other**	3	3	2	2	3	14
**Nausea**	3	1	3	0	1	8
**Vomiting**	1	1	2	0	1	5
**Fever**	1	2	1	0	0	4
**Hypertension**	0	0	0	0	1	1
**Hypersensitivity**	0	0	0	0	0	0
**Hypotension**	0	0	0	0	0	0

AE: Adverse event; Tx: treatment

In addition to the patient mentioned above, three other patients were removed from the study following the first treatment. Two patients developed suspect progressive disease: pericardial effusion in one and progressive liver lesions noted on abdominal ultrasound in the other. Both were euthanized prior to the second scheduled dose of doxorubicin and Immunocidin®. The third patient developed VCOG grade 3 hyporexia, grade 3 lethargy, and grade 4 nausea after treatment and it was elected to withdraw them from the study (Fig 2).

Two patients were removed from the study between the second and the third treatments (Fig 2). One patient developed metastatic disease and an alternative chemotherapy protocol was pursued. The second patient, previously mentioned in the materials and methods, developed VCOG grade 1 vomiting, grade 2 fever, grade 3 hyporexia and lethargy, and grade 5 diarrhea, thrombocytopenia, anemia. The patient was initially treated with oral maropitant, metronidazole, and subcutaneous fluids. After a lack of clinical improvement, the patient was hospitalized on IV fluids (LRS), IV metronidazole, IV maropitant, IV ondansetron, IV enrofloxacin, IV ampicillin sulbactam, IV buprenorphine, and oral capromorelin. Due to the severe anemia and thrombocytopenia, the patient received 3 units of canine packed red blood cells. Despite the aggressive supportive care, the patient died from complications of severe thrombocytopenia and anemia. The owner declined necropsy. Thus, the underlying cause of the severe thrombocytopenia and anemia are unknown.

Due to the development of these severe clinical signs, a mid-study AE analysis was completed on the enrolled patients at that time (n = 5). It was found that overall, the GI effect percentages (including nausea, vomiting, diarrhea, and hyporexia) were on average 2 times higher in the study population compared to what would be expected with doxorubicin alone [33]. In addition, all dogs (n = 5) experienced lethargy following the infusions of doxorubicin and Immunocidin®. One additional dog experienced VCOG grade 4 neutropenia and grade 3 thrombocytopenia, both of which resolved. Out of an abundance of caution, it was elected to reduce the starting dosage of doxorubicin for subsequent treatments and patients, and add additional prophylactic supportive medications, as outlined in the methods section.

One patient was removed from the study between doses three and four, after euthanasia due to suspect progressive disease: acute collapse with anemia and hypoproteinemia (Fig 2). Following the 5 doses of doxorubicin and Immunocidin®, two owners elected to give Yunnan Baiyao. No additional immunotherapy was reported.

### Outcome

At the end of the study, 16 patients had died and 2 were still alive. The median OST was 147 days (range: 39–668 days). Two patients still alive had a follow-up of 655 and 668 days, respectively. The first patient was an 11-year-old Labrador Retriever who initially presented for acute lethargy and panting. No evidence of metastatic disease was found on initial staging (stage II HSA; mitotic index: 12/10 hpf). He received all 5 treatment cycles and had intermittent VCOG grade 1–2 diarrhea that was metronidazole responsive. The second patient was a 10-year-old Wheaton Terrier who initially presented for a suspect seizure at home, vomiting, and lethargy. No evidence of metastatic disease was found on initial staging (stage II HSA; mitotic index: 1/10 hpf). Following the first treatment cycle, the patient developed VCOG grade 1 vomiting and diarrhea, grade 3 hyporexia, and grade 4 lethargy, nausea, neutropenia, and pyrexia. Due to these signs, the owners elected to withdraw the patient from the study. IV doxorubicin was continued without the Immunocidin® at a 25% reduced dosage, which was better tolerated. Following doxorubicin, the patient was started on metronomic cyclophosphamide.

### Statistical analysis

All 18 dogs were included in the statistical analysis with two dogs still being alive at data collection. The median OST and 95% confidence interval (CI) were estimated at 147 days (95% CI: 102–202), which was not statistically significantly different from historical controls (the 95% CI for this population overlaps with historical estimates: 107–257 days) [8, 32].

**Fig 4:**
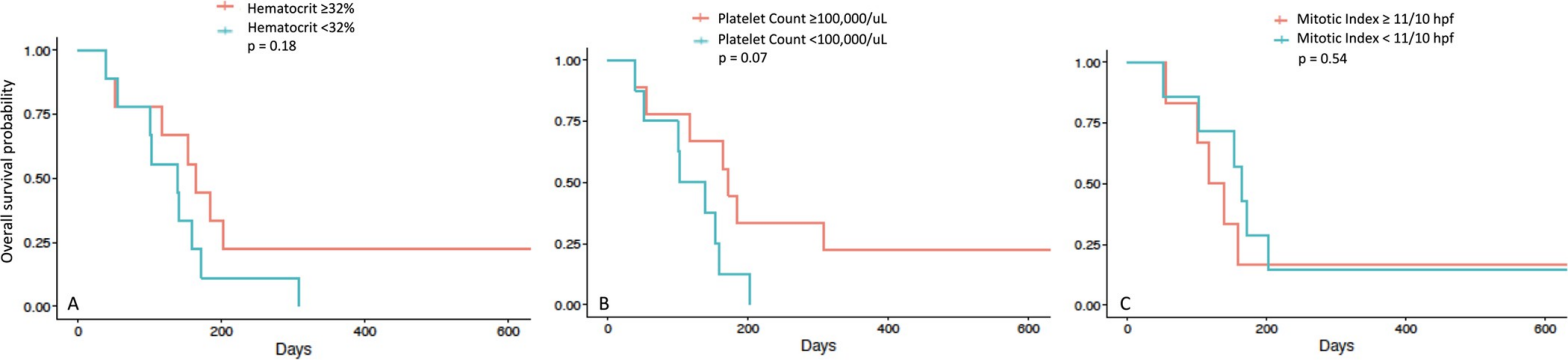
Hematocrit, platelet count, and mitotic index influence on survival time of dogs with splenic hemangiosarcoma. The threshold values used for dichotomization of each variable are median values. + Indicates patients who were censored.

Inspection of Schoenfeld residual plots and hypothesis testing for all 3 variables (anemia, thrombocytopenia, and mitotic index) confirmed the assumption of proportionality for the Cox proportional hazard model. No variable assessed was found to impact median OST (Fig 4). For the variable of anemia, the estimated hazard ratio of low vs. high hematocrit (<32% vs ≥32%) and its 95% CI was estimated at 1.99 (0.73–5.49) (p = 0.18). For the variable of thrombocytopenia, the estimated hazard ratio of low vs. high platelet count (<100,000/L vs ≥100,000/L) and its 95% CI was estimated at 2.72 (0.91–8.18) (p = 0.07). For the variable mitotic index, the estimated hazard ratio of low vs. high index (<11 mitoses/10 hpf vs ≥ 11 mitoses/10 hpf) and its 95% CI was estimated at 0.69 (0.20–2.30) (p = 0.54).

## Discussion

Immunocidin^®^ is a mycobacterial cell wall fraction derived from non-pathogenic *Mycobacterium phlei*. The two main immunomodulatory components of the mycobacterial cell wall fraction are mycolic acid and muramyl dipeptide. These components induce immune activation through a variety of mechanisms, stimulating both the direct and indirect immunity [18]. A mycobacterial cell wall fraction formulation has been used in human bladder cancer resistant to bacillus Calmette-Guérin, leading to a durable response in a small subset of patients [34]. In veterinary medicine, Immunocidin® is licensed for intratumoral injection of canine mixed mammary tumors and mammary adenocarcinoma. Two pilot studies evaluating its use IV in healthy dogs have been completed. In these studies, no clinically significant AEs were observed: the most common AE reported was mild to moderate transient hyperthermia. On necropsy, only a mononuclear inflammatory infiltration of the bronchioles was noted [19, 35]. IV Immunocidin® has been administered to dogs with osteosarcoma, transmissible venereal tumors, and multicentric lymphoma with varying degrees of success [35], and appears to be safe when combined with a variety of chemotherapy agents in tumor bearing dogs [18].

Reliable prognostic factors for canine HSA include tumor location, stage, and more recently, thrombocytopenia at diagnosis [7–10, 27, 36, 37]. Dogs with visceral HSA have a shorter survival time compared with those with dermal HSA, if treated with surgery alone (86 days vs 780 days) [9, 37]. Dogs with stage II or III HSA have been shown to have shorter survival times than those with stage I disease (62–210 days vs 345 days) [8–10]. Due to the small number of dogs in each category, the impact of stage on median OST within this population of patients could not be confirmed. Patients with thrombocytopenia have been shown to have shorter survival times compared to those with platelet counts within or above the reference interval, regardless of stage of disease [27]. The impact of anemia on outcome has been mixed, with some studies finding an impact on DFI [28], whereas other studies have not found anemia to be significantly associated with survival [27, 29]. It is difficult to compare evaluations across studies as the definitions of thrombocytopenia and anemia vary from study to study. Within the current study, thrombocytopenia was defined as a platelet count <100,000/uL, and anemia was defined as a hematocrit <32%. However, neither of these factors was found to have a statistical impact on OST. Results from a power analysis using the following parameters: (i) baseline event rate = 0.5, (ii) MST = 1.5, (iii) censoring rate = 0.3 and (iv) planned average length of follow-up = 5, with α = 0.05 and (1-β) = 0.80, suggest that a total of 126 and 56 dogs would be required to derive significant differences between low vs. high study groups for anemia and thrombocytopenia, respectively. Thus, a larger group of patients would be required to confirm the impact of anemia and thrombocytopenia on median OST for HSA patients.

Mitotic index has also been shown to impact survival, with survival times being significantly longer for patients with a lower mitotic index (those < 11 mitoses/10 hpf) vs. those with higher indices (≥ 11 mitoses/10 hpf) [5, 29]. Within this study, 13/18 patients had a mitotic index reported in a conventional way (per 10 hpf) to allow for comparisons. Within our patient population, mitotic index did not impact OST (p = 0.54), but larger numbers of patients are likely necessary to reveal a statistical impact of this parameter on survival.

Recently, the impact of body weight (in kilograms) was evaluated for impact on OST. It was found that the DFI was longer in patients weighing <20 kg, but this did not hold true for survival time, which was not impacted by patient size [38]. As there were only 2 patients under 20 kg in this group of patients, the impact of size on outcome could not be correlated.

With the clinically relevant increase in GI effects noted in the first 5 dogs enrolled, it was elected to decrease the starting dose of doxorubicin and increase the GI supportive medications administered concurrently. In this specific case, it was elected to decrease the dose of doxorubicin, as opposed to the Immunocidin®, due to the known propensity of doxorubicin to potentially cause severe GI AEs [33, 39], and the previous data that suggested IV Immunocidin® is generally well-tolerated in dogs [18]. However, it is impossible to know if these side effects were due to the doxorubicin, Immunocidin®, or the combination of the two drugs. If this combination is considered in the future, robust pharmacodynamic and pharmacokinetic studies should be completed.

Given that this was a pilot safety study, there are significant limitations that require mentioning. First, Immunocidin® is formulated as an emulsion using squalene, a natural lipid precursor to cholesterols and steroid hormones. With this formulation, Immunocidin® is USDA-licensed for intratumoral use in canine mammary tumors, while IV pharmacokinetic and pharmacodynamic evaluation has not been conducted in any species. Despite this lack of information, squalene has been shown to provide safe and effective drug delivery via parenteral routes, including IV [40, 41]. Preparation with squalene should prevent variations in distribution and elimination, which can occur with other emulsion formulations [42]. Also, at this time, work evaluating IV Immunocidin® is limited to published reports in equine and caprine species [22, 23], and international abstract presentations in dogs and cats [18–21].

Historical control comparisons are always difficult as patient populations are often varied between studies. Despite these limitations, the OST derived from this study (147 days) was not significantly different or improved from those reported previously with single agent doxorubicin (107–257 days) [8, 32]. In addition, one pathologist was not able to review all histologic specimens, and not all mitotic indices were reported in the same manner, limiting evaluation of the impact of mitotic index on median OST. Treatment was not always started within 14 days of splenectomy most commonly due to owner delay in treatment decisions. Delay of chemotherapy may have impacted OST, although true impact is unknown. Patients had stage II or III HSA, but there were small numbers in the stage III group, making assessment of the impact of disease stage on outcome impossible. In addition, not all patients were staged throughout or after treatment, rendering DFI difficult to define, and thus median OST (which is influenced by euthanasia decisions) was used as the primary endpoint instead.

Preliminary findings from this pilot study suggest that the combination of Immunocidin® and doxorubicin may cause more severe GI AEs when doxorubicin is administered at the maximum tolerated dosage, warranting a dosage reduction and aggressive prophylactic GI medications when the two medications are combined. In addition, based on our small cohort of dogs, there does not appear to be a survival advantage, or even a trend in improving survival compared to historical data, when doxorubicin and Immunocidin® are combined for canine splenic HSA at the dosages and treatment intervals utilized herein. Therefore, the results of the secondary aim of this study do not support subsequent clinical trials.

## Supporting information

S1 Data (CSV)
